# Changes in Gait Characteristics of Stroke Patients with Foot Drop after the Combination Treatment of Foot Drop Stimulator and Moving Treadmill Training

**DOI:** 10.1155/2021/9480957

**Published:** 2021-11-22

**Authors:** Chen Peishun, Zhou Haiwang, Li Taotao, Guan Hongli, Min Yu, Zhang Wanrong

**Affiliations:** Department of Rehabilitation Medicine, Guangzhou Panyu Central Hospital, China

## Abstract

**Objective:**

To study the changes in gait characteristics of stroke patients with foot drop after the combination treatment of foot drop stimulator and moving treadmill training and thus provide a basis for the improvement in a foot drop gait after stroke.

**Methods:**

Sixty patients with hemiplegia and foot drop caused by stroke were randomly divided into two groups of 30: the test group and the control group. Both groups received basic rehabilitation training. On this basis, the test group received the combination treatment of foot drop stimulator and moving treadmill training. The control group received foot drop stimulator training. Both groups received consecutive treatment for 3 weeks, five times a week, and every single time lasted for 30 minutes. Before and after the treatment, a gait watch three-dimensional gait analysis system was used to measure and record the maximum angles of flexion of the affected side's hip, knee, and ankle; the pace; the step length asymmetry; the iEMG of the tibialis anterior muscle; the functional ambulation category; and Ashworth's modified spasticity classification of the gastrocnemius.

**Results:**

After treatment, in the two groups, the maximum angles of flexion of the affected side's hip, knee, and ankle improved, the pace increased, the step length asymmetry decreased, the iEMG of the tibialis anterior muscle increased, the functional ambulation category improved, and Ashworth's modified spasticity classification of the gastrocnemius decreased, but the above changes in the test group were better than those in the control group. The difference is statistically significant (*p* < 0.05).

**Conclusions:**

The combination treatment of the foot drop stimulator and moving treadmill can significantly improve stroke patients' foot gait and promote the normalization of hip flexion, knee flexion, and ankle flexion. It can increase the pace, significantly reduce the step length asymmetry, reduce the muscle tone of the gastrocnemius, and improve walking function.

## 1. Introduction

A foot drop gait after stroke is very common, with an incidence rate as high as 20%-30% [1, 2], which manifests itself as ankle joints that cannot dorsiflex or can insufficiently dorsiflex, showing a cross-threshold gait when walking, affecting walking efficiency [3, 4].The ankle joint is the adjustment centre of human walking posture and stability. Its dorsiflexion function plays an important role in gait. Abnormal dorsiflexion function will affect the angle of hip flexion and knee flexion, which makes gait asymmetric and decreased the pace. Therefore, improving foot drop will help improve gait asymmetry and increase the pace. This study is to observe the effect of the combination treatment of foot drop stimulator and moving treadmill training on the foot drop gait after stroke and to provide a treatment basis for scientifically choosing the correct method for improving foot drop after stroke.

## 2. Methods

60 patients with hemiplegia and foot drop caused by stroke, who were treated in our hospital from January 2019 to March 2020, were selected and divided into the test group and the control group with 30 cases each. The inclusion criteria were as follows: (1) all patients met the diagnostic criteria for stroke in the “2016 Guidelines and Consensus on Diagnosis and Treatment of Chinese Cerebrovascular Diseases” [5] and were confirmed by cerebral CT or MRI as having cerebral infarction or cerebral haemorrhage; (2) stable vital signs, age from 30 to 70 years, and disease course from 15 days to 3 months; (3) hemiplegia in one limb and walking ability classification (functional ambulation category (FAC)) over or equal to grade 1; (4) Ashworth's modified spasticity classification of the gastrocnemius below or equal to grade 2 (if the muscle tone is greater than level 2, the foot drop stimulator cannot be used, so the muscle tone is below or equal to grade 2 here); and (5) no serious cognitive dysfunction. The exclusion criteria were as follows: (1) cerebellar stroke, (2) severe cognitive impairment, (3) four-limb paralysis, (4) Parkinson's disease, (5) lower limb fractures, (6) recent venous thrombosis of the lower limb, (7) recent myocardial infarction and congestive heart failure, and (8) those who have completed routine walking training. There were no statistical differences in gender, age, disease course, and stroke stratification between the two groups (Table 1). Since there is no significant difference in the number of cases between the two groups of ischemic stroke and haemorrhagic stroke, there is no difference in their impact on the results between them.

All patients received routine rehabilitation training, that is, neuromuscular promotion techniques; balance and standing training was given to the patients according to their functional status. On this basis, for foot drop, the test group received the combination treatment of the foot drop stimulator and moving treadmill; that is, walking training was carried out synchronously on the moving treadmill using the programmed electrical stimulation from the foot drop stimulator. The control group only received foot drop stimulator training. The treatment time of the two groups was 30 minutes/time, 5 times a week, for 3 consecutive weeks. This study was approved by the Research Ethics Committee of Guangzhou Panyu Central Hospital. All individuals provided written informed consent before participation in this study.

### 2.1. Foot Drop Stimulator

The XFT-2001 foot drop stimulator (XFT) is produced by Shenzhen Xunfengtong Electronics Co. Ltd. Before treatment, patients use a neuromuscular locator to locate the sensitive position on the tibialis anterior muscles. Then, patients can connect the main unit and use the walking mode. When the affected leg moves forward, the electrical stimulation is started to stimulate the tibialis anterior muscles to produce dorsiflexion. When the heel is on the ground, the electrical stimulation is turned off. With it, the patient can perform dorsiflexion of the foot in time during walking, and the patient's walking is more stable, more natural, and safer.

### 2.2. Moving Treadmill Training (MTT)

Moving treadmill training (MTT) is a task-oriented and supportive training. In active treadmill training, the patient walks at a set speed with the help of a flat track, just like walking in place. Without being restricted by space, the patients can exhibit a more symmetrical walking posture.

A gait watch three-dimensional gait analysis system was used to record the angles of flexion of the affected side's hip, knee, and ankle, before and after treatment. The pace, the step length asymmetry, the iEMG of the tibialis anterior muscle (with the help of Finland ME3000P surface electromyography, the integrated EMG value of the maximum contraction of the tibialis anterior muscle is measured, which measures the level of drop foot; the lower the iEMG value, the weaker the tibialis anterior muscle and the more serious the foot drop), and Ashworth's modified spasticity classification of the gastrocnemius of the two groups were also measured and recorded (Ashworth's modified spasticity classification is quantified; grade 1, grade 1+, grade 2, grade 3, and grade 4 were, respectively, recorded as 1, 1.5, 2, 3, and 4 grades. The higher the grade, the higher the muscle tone).

### 2.3. Outcomes and Statistical Analysis

The data obtained before and after the treatment was represented by “$\bar{x}\pm S$,” and the SPSS 22.0 statistical software package was used for data analysis. Comparison count data within the groups and between groups were tested by *χ*^2^. The measurement data were tested by *T*, and the test level was *α* = 0.05. *p* < 0.05 represents that the difference was statistically significant.

## 3. Results

**Figure 1:**
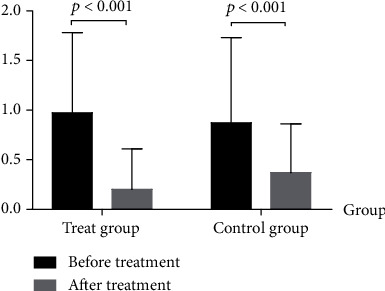
Ashworth's modified spasticity classification of the gastrocnemius.

**Figure 2:**
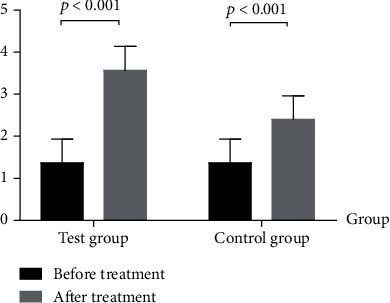
Comparison of two groups of FAC before and after treatment.

After treatment for 3 weeks, the data of the two groups including the maximum angles of flexion of the affected side's hip, knee, and ankle, the pace, the step length asymmetry, the FAC, the iEMG of the tibialis anterior muscle, and Ashworth's modified spasticity classification of the gastrocnemius all changed, but the above changes in the test group were better than those in the control group. The difference is statistically significant (*p* < 0.05, Tables 2 and 3 and Figures 1 and 2).

## 4. Discussion

After stroke, the patient is prone to an abnormal posture such as “circling gait” because of hemiplegia and foot drop, which will hinder the establishment of normal mobility mode and affect the recovery of lower limb motor function in hemiplegia [6]. The ankle joint is the adjustment centre of human walking posture and stability. Its dorsiflexion function plays an important role in gait. Abnormal dorsiflexion function will affect the angle of hip flexion and knee flexion, which increases step length asymmetry when walking, resulting in an abnormal gait such as foot sagging [7].

Foot drop after stroke is mainly caused by central nervous system injury [8]. Under normal circumstances, the dorsiflexion of the ankle joint is controlled by the common peroneal nerve. There are two branches of the common peroneal nerve, which control the anterior calf muscle group (tibialis anterior muscle) and lateral calf muscle group (long fibula and short muscle). The XFT-2001 foot drop stimulator stimulates the tibialis anterior and fibula long and short muscles by producing a reasonable and targeted electrical stimulation when patients lift their foot during walking, which will cause tibialis anterior and fibula long and short muscles to contract and make the ankle joint dorsiflex, thus correcting the foot drop and foot inversion [9]. The correction of foot inversion is helpful for the clearance of the swinging ankle joint during the gait cycle and reducing the body's compensatory posture [10]. The authors' previous research results also confirmed that the foot drop stimulator can help patients effectively improve their walking function and lower extremity motor function, reduce the step length asymmetry, and increase the pace [11]. However, the use of a foot drop stimulator requires patients to have a certain standing balance and walking ability and requires a sufficiently wide walking training field, which limits the use of the device.

Moving treadmill training is a task-oriented and supportive training. In the flat active training, the patient walks at a set speed on the flat track, just like walking in place, not limited by the venue and space. It can train the patient to develop a more symmetrical walking posture to improve walking speed and endurance [12]. Moving treadmill training can solve the problem of the patient's lack of balance function and the limitation of the training venue. In recent years, it has been more used for walking function training [13, 14]. However, if patients have a foot drop, when simply using moving treadmill to walk, they will be prone to sprain their ankle due to the constant speed of the crawler.

iEMG refers to the total amount of electrical activity generated by a muscle in a unit time, that is, the area enclosed by the electromyography curve and the time axis. When muscles contract statically at will, the value of iEMG is directly proportional to the size of muscle strength. Therefore, through the quantitative analysis of iEMG, the strength of the overall muscle activity can be determined. In this study, after treatment, the iEMG value of the tibialis anterior muscle from the test group was significantly increased, indicating that the recruitment of tibialis anterior motor units was increased, the motor activity was enhanced, and the muscle strength was restored. This was confirmed by the test results of the improvement of the maximum active dorsiflexion angle after treatment. With the recovery of the muscle strength of the ipsilateral tibialis anterior muscle, the maximum active dorsiflexion angle was increased, and the degree of foot drop during walking was correspondingly reduced. The improvement of foot drop made it easier for the patient to walk, so the walking performance was improved. Therefore, after treatment, the pace of the test group increased and the step length asymmetry decreased.

In this study, the combination treatment of foot drop stimulator and moving treadmill training made up for the shortcomings of the two methods above and fully combined their respective advantages. The results of the study showed that after treatment, the flexion angles of the affected side's hip, knee, and ankle of the test group changed significantly during the gait cycle. After treatment, the ankle dorsiflexion angle increased (*p* < 0.001), indicating that the motor function of the tibialis anterior muscle was restored and the muscle strength was enhanced, which benefited from the stimulation effect of the foot drop stimulator on the tibialis anterior muscle. The improvement of ankle dorsiflexion function and the driving of the movable flat track can help promote the flexion of the knee joint (normal 60°-70°) and hip joint (normal 20°-30°), so the flexion angles of the affected side's knee and hip increase after treatment (*p* < 0.001). With the normalization of the angles of hip flexion, knee flexion, and ankle flexion, the gait gradually tends to be symmetrical (after treatment, the step length asymmetry decreased), and the walking ability is also improved (after treatment, the FAC improved).

In this study, both groups of patients experienced decreased gastrocnemius muscle tone after treatment, which proved that the foot drop stimulator can help relieve the spasm. The muscle tone of the test group was significantly lower than that of the control group (*p* < 0.001), suggesting that walking on the MTT can make the patient more relaxed, and its combination with the foot drop stimulator is more helpful to reduce the muscle tone of the gastrocnemius muscles.

To sum up, the combination of foot drop stimulator and moving treadmill training can promote the recovery of lower limb motor function, increase the pace, decrease the step length asymmetry, improve the flexion angles of the affected side's ankle, knee, and hip, and reduce the gastrocnemius muscle tone, thereby improving the foot drop gait.

The shortcomings of this study are that the sample size of observation is small and the brain function test was not performed to observe the changes of the brain function area after intervention. The next step will increase sample size and use fMRI or fNIRS detection to further explore the neurokinetic mechanism of foot drop recovery after stroke.

## Figures and Tables

**Table 1:** Comparison of 2 groups of general information.

Team	*n*	Sex (*n*)		Age (year old)	Course of the disease (days)	Stroke stratification	
		Boy	Girl			Cerebral infarction	Cerebral hemorrhage
Test group	30	17	13	58.57 ± 8.47	44.37 ± 19.99	20	10
Control group	30	16	14	58.20 ± 7.98	44.23 ± 19.50	19	11
*X* ^2^/*t*		0.067		0.172	0.027	0.073	
*p*		0.795		0.864	0.979	0.787	

**Table 2:** Change of angle of hip flexion, knee flexion, and ankle flexion.

Team	*n*	Hip flexion (°)		Knee flexion (°)		Ankle flexion (°)	
		Before	After	Before	After	Before	After
Test group	30	10.93 ± 4.18	20.80 ± 3.62	30.97 ± 10.69	44.87 ± 13.16	4.57 ± 3.61	12.17 ± 3.43
Control group	30	10.73 ± 4.03	16.97 ± 4.29	30.90 ± 10.55	38.10 ± 11.06	4.23 ± 3.79	5.57 ± 4.05
*t*		0.189	4.293	0.024	2.156	0.349	6.808
*p*		0.851	0.000	0.981	0.035	0.729	0.000

**Table 3:** Change of the tibial anterior muscle iEMG, the pace, and the step length asymmetry.

Team	*n*	iEMG (*μ*V·s)		Pace (cm/s)		Step length asymmetry (cm)	
		Before	After	Before	After	Before	After
Test group	30	22.83 ± 18.04	60.83 ± 17.17	44.87 ± 7.98	60.03 ± 9.85	12.03 ± 3.20	4.60 ± 1.69
Control group	30	20.83 ± 17.27	39.00 ± 15.45	44.13 ± 7.84	47.47 ± 7.86	11.03 ± 3.11	9.30 ± 3.24
*t*		0.439	5.177	0.327	5.453	1.255	-7.016
*p*		0.663	0.000	0.745	0.000	0.215	0.000

## Data Availability

The data that support the findings of this study are available from the corresponding author upon request. The corresponding author is Chen Peishun (e-mail: cpshun21@126.com).

## Abbreviations

FAC: Functional ambulation category
XFT: XFT-2001 foot drop stimulator
MTT: Moving treadmill training
iEMG: Integral electromyography
fMRI: Functional magnetic resonance imaging
fNIRS: Functional near-infrared spectroscopy.
